# Is oral health–related quality of life of preschool children affected by the severity of early childhood caries?

**DOI:** 10.3389/froh.2026.1730928

**Published:** 2026-02-23

**Authors:** Abrar Alanzi, Sahar Behzadi, Aishah Alsumait, Jagan Baskaradoss

**Affiliations:** 1 Department of Developmental and Preventive Sciences, College of Dentistry, Kuwait University, Sabah Al Salem University City, Kuwait City, Kuwait; 2 Kuwait Ministry of Health, Sulaibikhat, Kuwait

**Keywords:** caries severity, early childhood caries (ECC), oral health related quality of life (OHRQOL), preschool, PUFA index

## Abstract

**Objectives:**

This national cross-sectional study investigated the severity of early childhood caries (ECC) and its impact on the oral health–related quality of life (OHRQoL) of preschool children and their families, providing large-scale population-based evidence.

**Methods:**

Kindergarten children (levels I and II) from randomly selected schools across all six governorates were examined. Caries experience was recorded using dmft/dmfs and merged ICDAS criteria, and caries severity was assessed using the pufa index. Demographic data and OHRQoL were obtained from caregivers using the validated Arabic version of the Early Childhood Oral Health Impact Scale (A-ECOHIS).

**Results:**

Of 1,783 examined children, 892 caregivers completed the questionnaire. ECC prevalence was 88.6%, with a mean dmft of 6.45 ± 4.5 and dmfs 13.0 ± 14.1. Extensive lesions (ICDAS 5–6) were observed in 63.4%, and 9.3% showed clinical consequences of untreated caries (PUFA > 0). Caries experience was significantly associated with higher child- and family-level A-ECOHIS scores (*p* < 0.001). Clinical consequences of untreated caries (pufa) were associated with poorer child-level OHRQoL, but not family-level OHRQoL. Lower paternal education and higher birth order were associated with greater caries experience (*p* < 0.05).

**Conclusions:**

Early childhood caries experience was strongly associated with poorer oral health–related quality of life among preschool children and their families. ECC severity was associated with poorer child-level OHRQoL but showed no additional impact at the family level. Early, family-centered preventive strategies are essential to reduce the burden of ECC and enhance child well-being.

## Introduction

Early childhood caries (ECC) is recognized as a significant and prevalent oral health issue among young children, aged under six years (1). The estimated prevalence of ECC was 49% globally and 72% in the Middle East (2). If left untreated, dental caries can have adverse effects on a child's development. It may cause oral health–related quality of life (OHRQoL) issues, such as dental pain, difficulty chewing, poor appetite, weight loss, sleep disturbances, and low academic performance (3–7).

Several indices are used to describe the dental caries status at the national and community level. In oral epidemiological studies, dental caries is commonly evaluated by the DMFT and ICDAS indices (8). The DMFT/dmft index is a World Health Organization criterion that measures the number of decayed, missing, and filled teeth. The International Caries Detection and Assessment System (ICDAS) is used to detect dental caries at different clinical stages, including early non-cavitated enamel lesions (9). However, neither index provides insights into the oral implications of left untreated caries. To address this issue, Monse et al. introduced the pufa/PUFA index in 2010, which specifically assesses four oral consequences of advanced untreated carious lesions, including exposed pulp, oral mucosal ulceration due to remaining roots, fistulas, and abscesses (10).

In populations with a high prevalence of dental caries among preschool children, a comprehensive understanding of oral health status, including disease severity and its impact on overall health and quality of life, is essential. Although few studies across different populations have reported the negative impact of early childhood caries (ECC) on preschool children's oral health–related quality of life (OHRQoL) (11–16), they were limited by small or region-specific samples. In addition, the existing evidence remains heterogeneous in assessing caries severity and in evaluating the clinical consequences of untreated caries (15). Moreover, nationally representative data integrating comprehensive clinical indices with validated OHRQoL measures are scarce. This large-scale national cross-sectional study investigated the clinical consequences of untreated ECC and their influence on the OHRQoL of preschool children and their families using a validated Arabic OHRQoL instrument (A-ECOHIS). It was hypothesized that greater caries experience and severity, including untreated clinical consequences, would be associated with poorer OHRQoL outcomes. The findings provide strong national evidence that contributes to global understanding of the ECC burden and support the development of preventive and clinical strategies to improve oral health outcomes in preschool children.

## Materials and methods

The report of this study was developed following the STROBE Statement checklist for cross-sectional studies.

### Study design

This is a cross-sectional national study conducted in randomly selected kindergarten schools across all six governorates in Kuwait. Data collection was carried out over 6 months during the 2022/2023 academic year. Ethical approval was obtained from the Ethical Committee of the Health Sciences Centre, Kuwait University, and the Ministry of Health. This study was a collaborative research between Kuwait University and the School Oral Health program, Ministry of Health. Permission to conduct the study was also obtained from the Ministry of Education Research Department and the school principals. Detailed explanatory letters outlining the aims of the study and informed consent forms were given to the parents of each student through their class teachers. The inclusion criteria included healthy, cooperative Kuwaiti children aged 4–5 years and signed consent forms from parents/guardians. Exclusion criteria included noncooperative children and those with any systemic disease.

### Study group

According to the most recent data from the Statistics Service System in Kuwait, there are around 4,000–11,000 Kuwaiti kindergarten children in each governorate. The total number of classes per governorate ranges from 110 to 200, with a mean student-to-class ratio of 20–30. The sampling design was a cluster sampling. The exploratory method defined by the World Health Organization (WHO) (17) would be used to determine the number of subjects required per governorate. It was recommended to select 20–50 subjects from each cluster to assess the prevalence of a disease. Initially, two schools were randomly selected from each of the six governorates. However, to achieve the desired sample size and account for expected non-response and absenteeism, the number of participating schools was increased to 22 randomly selected schools across all governorates. From each selected school, one class was randomly chosen. Based on a previously reported ECC prevalence of 70% among Kuwaiti preschool children (11), the minimum required sample size was estimated at 480 children. To compensate for the cluster design and incomplete responses, the final target sample was increased to approximately 800 children. To ensure proportional representation across all six governorates, the required number of children from each governorate was estimated based on the relative distribution of kindergarten children within the total population. Population data for the 2022–2023 academic year were obtained from the Ministry of Education, and the sample size for each governorate was allocated proportionally to the governorate's share of the total kindergarten population. Based on this proportional allocation, the required number of participating schools and children from each governorate was determined. This proportional allocation approach ensured adequate geographic representation and minimized sampling bias at the national level.

The predetermined number of schools from each governorate was selected from the sampling frame using computer-generated random numbers, in accordance with WHO oral health survey recommendations (17). The updated list of classes and the number of students in each class were obtained from each school. The required number of students from each grade was selected from this list. A consent form was sent to the parents of the selected students a week before the school visit, and only those students who had a signed consent form from their parents were examined.

### Study procedure

#### Clinical examination

All clinical examinations were performed at the school premises (nurse's room) by six trained and calibrated general dentists. Examinations were conducted using reclining chairs and portable lights (Sun-Led Classic, BPR Swiss, Switzerland), and mouth mirrors without explorer or compressed air. A periodontal probe and gauze were used to remove any debris or excess food covering the teeth. Each calibrated examiner was accompanied by a trained chair-side assistant.

All dental surfaces were examined, and dental caries was diagnosed visually according to the WHO indices (decayed, missing, and filled surfaces (dmfs) and teeth (dmft) and ICDAS-II criteria (17, 18). The caries experience was classified as: caries-free (dmft = 0) and caries-positive (dmft > 0). For the evaluation of the child with a single ICDAS score, among all other scores of teeth, the maximum score was categorized as “initial” (ICDAS 1–2), “moderate” (ICDAS 3–4), or “extensive decay” (ICDAS 5–6).

The pufa index was used to evaluate the clinical consequences of untreated dental caries in primary teeth (10). Lesions in the surrounding tissues not related to a tooth, with detectable pulpal involvement due to caries, were not recorded. The assessment was made visually with no use of any instrument. Only one score was assigned per tooth. The pufa index criteria are regarded as:

p: Pulpal involvement, observable pulp chamber, carious coronal tooth structure, and remaining roots or root fragments.

u: Ulceration as a result of a sharp tooth piece (dislocated tooth with pulpal involvement or root fragments).

f: Fistula, a pus-discharging sinus tract associated with a primary tooth.

a: Abscess, a swelling associated with a primary tooth.

The pufa count was computed as (p  + u + f + a) per child. The count range in the primary dentition was from zero to 20.

### Calibration

Calibration of the participating examiners, consisting of three female and three male general dentists who were registered dental practitioners, was conducted against a reference examiner (AA) who served as the gold standard. The procedure consisted of an initial theoretical session that included practical exercises using clinical images and extracted carious primary teeth, followed by a clinical session in which the trainees and the reference examiner examined patients together. In the clinical part of the calibration process, each participating examiner and the reference examiner examined 10 children (not included in the study sample), followed by reexamination at a 1-week interval. The reference standard examiner (AA) is an experienced and calibrated pediatric dentist. The intra-examiner reliability kappa score for the reference examiner exceeded 0.9. During the whole calibration session, the information technology team was available to perform the analysis simultaneously so that repeated cycles of testing and training of the examiners could be carried out until all of them reached at least satisfactory levels of performance (>0.80). All examiners achieved satisfactory inter-examiner reliability, with Cohen's kappa values ≥ 0.80 for dmft, ICDAS, and PUFA indices.

After the examination, all participant children received incentives (stickers) and fluoride varnish if it had not been applied in the last 6 months. All parents/caregivers were provided with data on their children's oral health status, and if their child had carious lesions or disease complications, they were informed to seek dental treatment services.

#### Questionnaire

A structured questionnaire was used to gather information from the parent/caregiver. The following information was obtained: demographic information related to the child (child's gender, age, and child rank), and demographic information related to the parent (marital status, age of mother, and educational level of parents). OHRQoL of children was assessed using the Early Childhood Oral Health Impact Scale, ECOHIS (19). The ECOHIS has shown good reliability and validity (20–23). The ECOHIS consists of 13 questions split into two parts: the child impact section and the family impact section. The child part has nine items related to four areas: symptoms, function, psychology, and child self-image/ social interaction. The family section contains four items across two areas: parental distress and family function. The Arabic version of ECOHIS was formulated and validated (24). It is reliable to utilize for Arabic-speaking parents of kindergarten children.

The A-ECOHIS questionnaire is counted using an easy 6-point Likert scale. The responses were coded as follows: never = 0, hardly ever = 1, occasionally = 2, ofte*n* = 3, very often = 4, and don't know = 5. The total count was calculated by adding the scores of all questions. The child part scores range from 0 to 36, and the family part scores range from 0 to 16. A high total score indicates a major effect and further complications. The response “don't know” was handled as a missing value, as described in the original study [Pahel et al., (19)]. The exclusion criteria included incomplete answers or answers with “dońt know” in two items or more, either in child domains or family domains. The A-ECOHIS, together with the questionnaire, was given to the participating parents/caregivers for completion through their children's class teacher.

#### Statistical analysis

Data were managed and analyzed using the Statistical Package for the Social Sciences (SPSS for Windows, version 26.0; SPSS Inc., Chicago, Ill., USA). Descriptive statistics (mean, frequencies, percentage) were performed. A Chi-square test was used for nominal or ordinal variables. Data normality was tested using the Kolmogorov–Smirnov test. Differences in the mean values of the indices were assessed using the Mann–Whitney and Kruskal–Wallis nonparametric tests. Cohen's kappa coefficient (*κ*) was used to assess the intra-examiner and inter-examiner reliability regarding the scoring indices.

The association between the dependent variable (overall ECOHIS scores) and the independent variables [child factors (sex, age, dmft, pufa) and parental factors (father's education level, mother's education level)] was tested using a Poisson regression model. Independent variables with *p*-values ≤ 0.20 in the bivariate analysis were included in the multivariable model. Significant independent variables (*p* value < 0.05) were selected for the final model (25). The level of statistical significance was set at 0.05.

## Results

**Figure 1:**
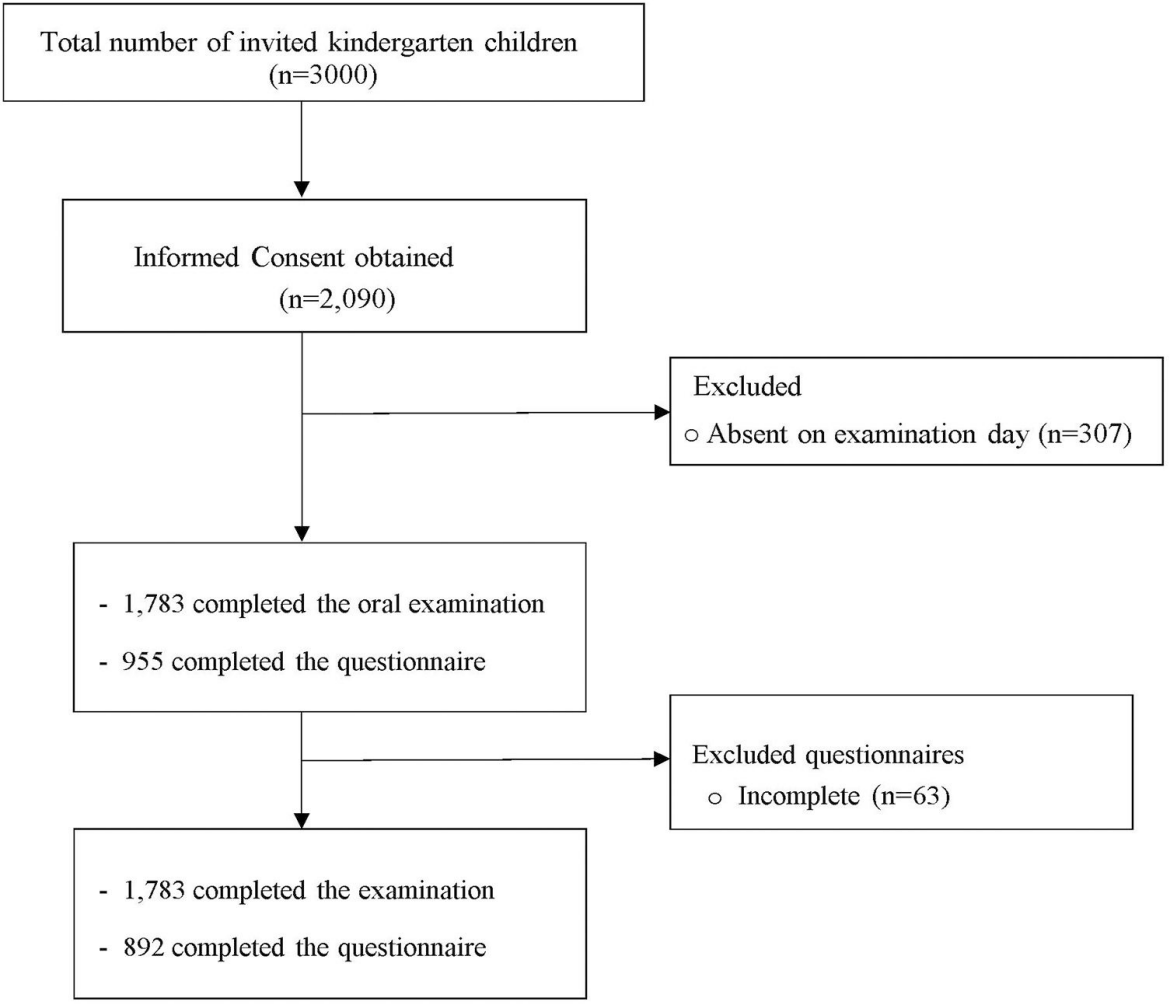
Flow chart showing participant recruitment, exclusions, and final samples included.

A total of 3,000 kindergarten children enrolled in 22 randomly selected schools across Kuwait's six governorates were invited to participate in the study. Written informed consent forms were obtained from the parents or caregivers of 2,090 children (69.7%). On the scheduled examination days, 307 children were absent. Consequently, 1,783 children received the complete oral examination and were included in the final analysis. A total of 955 questionnaires were collected. Of those, 63 questionnaires had incomplete responses and were excluded, resulting in 892 children who completed both the examination and the questionnaire (Figure 1).

Table 1 presents the demographic characteristics and oral health variables of all participating children (*n* = 1,783). Of the total sample, 764 were in KG1 (48–59 months), and 1,019 were in KG2 (60–71 months). The gender distribution was nearly equal, with 48.6% males and 51.4% females. The overall caries prevalence was 88.6%, with mean dmft = 6.45 ± 4.5 and mean dmfs = 13.0 ± 14.1. Untreated caries constituted the majority of dmft (dt = 5.68 ± 4.1). Extensive lesions (ICDAS 5–6) were the predominant type (63.4%), and 9.3% of children exhibited clinical consequences of untreated caries, with pulp involvement in 4.8% and abscesses in 3.5% (Tables 1, 2).

**Table 1:** Demographic features and oral health variables of the participant children.

Variables	Kg1 (48–59 mos) *N* = 764 (%)	Kg2 (60–71 mos) *N* = 1,019 (%)	Total *N* = 1,783
Governate			
Al-Ahmadi	198 (25.9)	193 (18.9)	391 (21.9)
Al-Asima	82 (10.7)	129 (12.7)	211 (11.8)
Al-Jahra	151 (19.7)	226 (22.2)	377 (21.2)
Mubrak Al-Kabeer	162 (21.2)	148 (14.5)	310 (17.4)
Hawally	92 (12.0)	109 (10.7)	201 (11.3)
Al-Farwanyiah	79 (10.3)	214 (21.0)	293 (16.4)
Gender			
Male	377 (49.4)	490 (48.1)	867 (48.6)
Female	387 (50.6)	529 (51.9)	916 (51.4)
Caries			
Yes	656 (85.9)	923 (90.6)	1,579 (88.6)
No	108 (14.1)	96 (9.4)	204 (11.4)
ICDAS			
mild _d1−2_	91 (11.9)	89 (8.7)	180 (10.1)
moderate _d3−4_	147 (19.3)	120 (11.8)	267 (15.0)
extensive_d5−6_	418 (54.7)	714 (70.0)	1,132 (63.4)
pufa	53 (6.9)	113 (11.1)	166 (9.3)
pulp	30 (3.9)	56 (5.5)	86 (4.8)
ulcer	3 (0.4)	12 (1.2)	15 (0.8)
fistula	8 (1.0)	13 (1.3)	21 (1.2)
abscess	18 (2.4)	45 (4.4)	63 (3.5)

Descriptive statistics are presented as frequencies and percentages, mos, months.

**Table 2:** Difference in dmft, dmfs and pufa indices among participant children.

Variables	Kg1	Kg2	Total
	(48–59 mos)	(60–71 mos)	
	Mean (SD)	Mean (SD)	Mean (SD)
dmft	5.63 (4.3)	7.06 (4.5)	6.45 (4.5)
dt	5.14 (3.9)	6.09 (4.1)	5.68 (4.1)
mt	0.16 (0.8)	0.37 (1.1)	0.28 (0.9)
ft	0.34 (1.1)	0.60 (1.4)	0.49 (1.3)
dmfs	10.22 (10.1)	15.1 (14.8)	13.0 (14.1)
ds	7.90 (9.1)	10.26 (10.4)	9.25 (9.9)
ms	0.81 (4.1)	1.85 (5.3)	1.40 (4.9)
fs	1.54 (5.1)	2.99 (7.1)	2.37 (6.4)
pufa	0.1 (0.4)	0.15 (0.5)	0.13 (0.4)
pulp	0.05 (0.3)	0.07 (0.3)	0.06 (0.3)
ulcer	0 (0.1)	0.02 (0.2)	0.01 (0.1)
fistula	0.01 (0.1)	0.01 (0.1)	0.01 (0.1)
abscess	0.03 (0.2)	0.05 (0.2)	0.04 (0.2)

Differences between groups were assessed using the Mann–Whitney *U*-test.

mos, months; ds, decayed surfaces; ms, missed surfaces; fs, filled surface; dmfs, decayed, missed, filled surfaces; dmft, decayed, missed, filled teeth.

Table 3 summarizes the characteristics of the 892 children for whom both clinical and caregiver questionnaire data were available. Of these, 382 were in KG1 and 510 in KG2, with an almost equal gender distribution (50.1% male, 49.9% female). Approximately 18.7% were first-born children, and 23.1% second-born. One-fifth of the children (20.2%) were in the fifth or later birth order. Most fathers (48.8%) and mothers (70.0%) held a diploma/college degree, and only a small proportion of parents had postgraduate education (fathers = 6.8%; mothers = 3.1%). The majority of mothers were aged 25–45 years (95.5%). Almost all parents were married (92.9%). Low father's education was significantly associated with high children's dmft scores (*p* = 0.035). In contrast, low mother's education was not associated with dmft scores but was significantly related to high pufa scores (*p* = 0.043). Childbirth order (rank) was associated with both dmft (*p* = 0.005) and pufa (*p* = 0.034), with later-born children having higher caries experience and severity. No significant differences were observed by gender.

**Table 3:** Demographic features of the participant children and caregivers.

Variables	Kg1	Kg2	Total
	(48–59 mos)	(60–71 mos)	
	*N* = 382 (%)	*N* = 510 (%)	*N* = 89
Gender			
Male	205 (53.7)	242 (47.5)	447 (50.1)
Female	177 (46.3)	268 (52.5)	445 (49.9)
Child rank			
First child	77 (20.2)	90 (17.6)	167 (18.7)
Second child	83 (21.6)	123 (24.1)	206 (23.1)
Third child	85 (22.3)	116 (22.8)	201 (22.5)
Fourth child	55 (14.4)	83 (16.3)	138 (15.5)
Fifth and more	82 (21.5)	98 (19.2)	180 (20.2)
No. of siblings			
None	9 (2.4)	22 (4.3)	31 (3.5)
One	61 (16.0)	61 (12.0)	122 (13.7)
Two	100 (26.2)	104 (20.4)	204 (22.9)
Three	80 (20.9)	127 (24.9)	207 (23.2)
Four	56 (14.7)	82 (16.1)	138 (15.5)
Five and more	76 (19.9)	114 (22.3)	190 (21.3)
Father's education			
Less than high school	55 (14.4)	90 (17.6)	145 (16.3)
High school	117 (30.6)	134 (26.3)	251 (28.1)
Diploma/College	185 (48.4)	250 (49.0)	435 (48.8)
Master/PhD	25 (6.5)	36 (7.1)	61 (6.8)
Mother's education			
Less than high school	20 (5.2)	39 (7.6)	59 (6.6)
High school	78 (20.4)	103 (20.2)	181 (20.3)
Diploma	272 (71.3)	352 (69.1)	624 (70.0)
Master/PhD	12 (3.1)	16 (3.1)	28 (3.1)
Mother's age			
Less than 25	12 (3.1)	12 (2.4)	24 (2.7)
25–35	240 (62.8)	292 (57.3)	532 (59.6)
36–45	125 (32.7)	195 (38.2)	320 (35.9)
More than 45	5 (1.4)	11 (2.1)	16 (1.8)
Marital Status of Parents			
Married	358 (93.7)	471 (92.4)	829 (92.9)
Divorced/widow	24 (6.3)	39 (7.6)	63 (7.1)

Descriptive statistics are presented as frequencies and percentages.

Based on the 892 valid A-ECOHIS responses (Tables 4, 5), dental pain was the most frequently reported child impact (54.9%), followed by eating difficulties (13.8%), irritability (19.9%), and trouble sleeping (11.5%). Avoidance of smiling (8.0%) and reduced social interaction (7.9%) were less common. At the family level, 34.1% of caregivers reported being upset, 33.1% reported feelings of guilt, and 36.3% had to take time off work due to their child's oral condition. Financial burdens were less frequently reported (17.8%). Children with caries (dmft ≥ 1) exhibited significantly higher ECOHIS scores than caries-free peers (child impact mean 7.65 vs. 4.88, *p* = 0.02; family impact mean 3.89 vs. 1.65, *p* < 0.001). Although children with PUFA ≥ 1 and extensive ICDAS lesions showed higher mean ECOHIS scores, these differences did not reach statistical significance in the unadjusted analyses (Table 6).

**Table 4:** Responses to the arabic early childhood oral health impact scale (A-ECOHIS)—children and their caregivers (*n* = 892).

Impact	ECOHIS response, *n* (%)					Mean (SD)
	Never	Hardly ever	Occasionally	Often	Very often	
Child impact section						7.32 (26.4)
a. Had pain in the teeth, mouth, or jaws?	402 (45.1)	221 (24.8)	217 (24.3)	38 (4.3)	9 (1.0)	1.21 (4.12)
b. Had difficulty drinking hot or cold beverages	642 (72.0)	133 (14.9)	87 (9.8)	18 (2.0)	1 (0.1)	1.09 (6.08)
c. Had difficulty eating some foods	647 (72.5)	123 (13.8)	91 (10.2)	19 (2.1)	5 (0.6)	0.86 (4.88)
d. difficulty pronouncing any words	616 (69.1)	112 (12.6)	119 (13.3)	29 (3.3)	12 (1.3)	0.79 (3.76)
e. Missed preschool, daycare, or school	535 (60.0)	184 (20.6)	149 (16.7)	17 (1.9)	5 (0.6)	0.74 (2.72)
f. Had trouble sleeping	703 (78.8)	100 (11.2)	72 (8.1)	10 (1.1)	3 (0.3)	0.57 (3.72)
g. Been irritable or frustrated	582 (65.2)	127 (14.2)	126 (14.1)	35 (3.9)	18 (2.0)	0.87 (3.77)
h. Avoided smiling or laughing	714 (80.0)	101 (11.3)	49 (5.5)	18 (2.0)	4 (0.4)	0.67 (4.53)
i. Avoided talking with other children	701 (78.6)	108 (12.1)	53 (5.9)	24 (2.7)	3 (0.3)	0.52 (3.25)
Family impact section						3.63 (15.5)
How often have you or another family member have ………. because of your child's dental problems or treatment?						
a. Been upset	588 (65.9)	123 (13.8)	125 (14.0)	33 (3.7)	18 (2.0)	0.92 (4.18)
b. Felt guilty	597 (66.9)	120 (13.5)	97 (10.9)	49 (5.5)	21 (2.4)	1.10 (5.23)
c. Had taken time off from work	568 (63.7)	127 (14.2)	153 (17.2)	29 (3.3)	8 (0.9)	1.05 (4.89)
d. Had a financial impact on your family	733 (82.2)	70 (7.8)	62 (7.0)	12 (1.3)	11 (1.2)	0.55 (3.74)

*Significant *P* < 0.05.

**Table 5:** Responses to the arabic early childhood oral health impact scale (A-ECOHIS) in regard to caries experience and caries severity.

Impact	Caries Experience (dmft)		Caries Severity (pufa)		ICDAS			
	dmft=0 (*n* = 107)	dmft≥1 (*n* = 785)	pufa=0 (*n* = 846)	pufa≥1 (*n* = 46)	Sound (Score 0)	Initial (Scores 1 & 2)	Moderate (Scores 3 & 4)	Extensive (Scores 5 & 6)
	Mean (SD)	Mean (SD)	Mean (SD)	Mean (SD)	Mean (SD)	Mean (SD)	Mean (SD)	Mean (SD)
Child impact section								
a. Had pain in the teeth, mouth, or jaws?	0.43 (0.70)	1.32 (4.41)*	1.18 (4.23)	1.70 (1.07)*	0.49 (0.75)	1.97 (9.06)*	0.68 (0.90)	1.38 (4.02)
b. Had difficulty drinking hot or cold beverages	0.79 (5.33)	1.13 (6.17)	1.03 (5.95)	2.07 (8.04)	0.75 (5.08)	1.76 (9.08)	1.14 (6.81)	1.06 (5.61)
c. Had difficulty eating some foods	0.80 (5.32)	0.87 (4.82)	0.79 (4.65)	2.07 (8.05)	0.79 (5.08)	1.83 (9.08)	0.73 (4.86)	0.78 (4.03)
d. difficulty pronouncing any words	0.90 (5.34)	0.78 (3.49)	0.78 (3.85)	0.93 (1.29)	0.89 (5.09)	1.32 (6.46)	0.54 (0.98)	0.76 (3.35)
e. Missed preschool, daycare, or school	0.47 (0.79)	0.78 (2.89)*	0.74 (2.78)	0.76 (0.87)	0.99 (5.08)	1.22 (6.49)	0.57 (0.84)	0.67 (0.88)
f. Had trouble sleeping	0.24 (0.66)	0.61 (3.96)*	0.57 (3.82)	0.54 (0.89)	0.74 (5.09)	1.00 (6.49)	0.16 (0.44)	0.57 (3.31)
g. Been irritable or frustrated	0.54 (0.96)	0.91 (4.00)*	0.87 (3.86)	0.89 (1.10)	1.09 (5.11)	2.10 (9.06)*	0.51 (0.97)	0.75 (2.48)
h. Avoided smiling or laughing	0.32 (0.77)	0.72 (4.82)*	0.68 (4.65)	0.48 (0.75)	0.83 (5.10)	1.07 (6.49)	0.66 (4.86)	0.59 (4.01)
i. Avoided talking with other children	0.38 (0.83)	0.54 (3.45)	0.52 (3.33)	0.54 (0.86)	0.85 (5.09)	1.06 (6.49)	0.27 (0.63)	0.44 (2.40)
Family impact section								
How often have you or another family member have ………. because of your child's dental problems or treatment?								
a. Been upset	0.50 (0.89)	0.97 (4.44)*	0.91 (4.28)	0.98 (1.37)	0.96 (5.10)	1.22 (6.49)	0.95 (4.89)	0.86 (3.37)
b. Felt guilty	0.47 (0.91)	1.19 (5.56)*	1.03 (5.03)	2.43 (8.05)	0.94 (5.11)	1.07 (6.49)	0.89 (4.89)	1.19 (5.17)
c. Had taken time off from work	0.47 (0.83)	1.13 (5.20)*	1.06 (5.02)	0.87 (0.96)	0.46 (0.81)	1.14 (6.48)	0.99 (4.89)	1.17 (5.15)
d. Had a financial impact on your family	0.21 (0.63)	0.60 (3.97)*	0.56 (3.83)	0.50 (1.03)	0.25 (0.69)	0.93 (6.49)	0.66 (4.87)	0.54 (3.33)

Differences in means were assessed using the Mann–Whitney *U*-test or Kruskal–Wallis test, as appropriate.

* Significant *P* < 0.05.

**Table 6:** The association between the difference in caries experience, pufa scores, ICDAS and A-ECOHIS sections.

Variables	N (%)	Mean A-ECOHIS score (SD)			
		Child Level		Family Level	
		Mean (SD)	*P* value	Mean (SD)	*P* value
Caries Experience					
None	107 (12.0)	4.88 (10.2)	0.02*	1.65 (2.6)	<0.001*
1 or more	785 (88.0)	7.65 (27.8)		3.89 (16.5)	
PUFA					
None	846 (94.8)	7.17 (26.8)	0.14	3.56 (15.8)	0.41
1 or more	46 (5.2)	9.98 (16.4)		4.78 (9.3)	
ICDAS					
Sound	118 (13.2)	7.42 (27.1)	0.203	2.61 (10.3)	0.868
Initial	72 (8.1)	13.33 (63.2)		4.36 (25.8)	
Moderate	129 (14.5)	5.26 (11.2)		3.49 (19.4)	
Extensive	573 (64.2)	7.01 (20.1)		3.77 (13.6)	

Comparisons of mean A-ECOHIS scores were performed using the Mann–Whitney *U*-test or Kruskal–Wallis test, as appropriate.

* Significant *P* < 0.05.

Multivariable Poisson regression analysis was conducted to examine factors independently associated with child- and family-level OHRQoL after adjustment for potential confounders (Table 7). Caries experience was significantly associated with higher child and family A-ECOHIS scores, indicating poorer oral health–related quality of life (*p* < 0.001). However, pufa scores were only significantly associated with higher child A-ECOHIS scores, In the adjusted regression model, lower father's educational level, higher birth order, younger child age, and male gender were associated with increased child and family A-ECOHIS scores (*p* < 0.01).

**Table 7:** Multivariate poisson regression model for overall scores of A-ECOHIS scores (child and family) and clinical factors (caries experience, pufa scores, ICDAS) after adjusting for the selected socio-demographic variables (age, gender, birth order, father and mothers' education).

Variables	Child Level		Family Level	
	Adjusted RR (95% CI)	*P* value	Adjusted RR (95% CI)	*P* value
Caries Experience				
None	Ref		Ref	
1 or more	4.82 (4.21– 5.52)	<0.001	4.79 (3.81–6.01)	<0.001
PUFA				
None	Ref		Ref	
1 or more	1.27 (1.15–1.40)	<0.001	1.02 (0.89–1.18)	0.74
ICDAS				
Sound	Ref		Ref	
Initial	0.35 (0.28–0.52)	<0.001	0.54 (0.44–0.66)	<0.001
Moderate	0.21 (0.19–0.24)	<0.001	0.40 (0.33–0.49)	<0.001
Extensive	0.27 (0.24–0.30)	<0.001	0.40 (0.34–0.49)	<0.001

Model: Caries experience, PUFA, ICDAS, age, Gender, Child birth order, Father education, Mother education Multicollinearity was assessed prior to model construction and was within acceptable limits.

## Discussion

The present national study provides new evidence on the burden, severity, and clinical consequences of early childhood caries (ECC), as well as its impact on the oral health–related quality of life (OHRQoL) of preschool children and their families. With nearly 9 out of 10 children affected, the prevalence of ECC in this study is among the highest reported globally. The findings reaffirm that ECC remains a major global public health concern, continuing to affect children's overall health and development (10–16).

The high prevalence of ECC, reflected in the high decayed component of the dmft index, indicates insufficient preventive care and early intervention. This finding is consistent with reports from other high-prevalence populations and reflects the continuing global differences in access to preventive and restorative dental care (15, 26–32). The high proportion of extensive lesions (ICDAS 5–6) and the presence of clinical consequences measured by the pufa index demonstrate that untreated caries remains a neglected condition that can lead to infection, pain, and impaired nutrition. Our findings were consistent with previous international reports linking severe dental caries to poor growth and lower body mass index in children (2, 10, 29).

The results of this study confirmed the profound psychosocial problem of ECC. Children with greater caries experience exhibited significantly poorer child and family OHRQoL. However, the contribution of advanced clinical consequences was more evident at the child level than at the family level. Caregivers of the affected children reported higher emotional distress and work disruption. These findings align with numerous studies demonstrating that dental caries negatively affects children's daily performance, eating and sleeping patterns, and social confidence (15, 26–32). The impact on caregivers highlights the broader family aspect of oral diseases, which has increasingly been recognized as an integral component of comprehensive child health (5, 15). The findings support the FDI's global framework that defines oral health as an integral part of overall health and quality of life (33).

From a public health perspective, the associations found between demographic factors and caries experience in this study reflect broader determinants of oral health disparities seen globally. The significant relationship between lower paternal education, higher birth order, and greater caries severity is consistent with findings reported from different populations (34–36). These consistent results suggest that socioeconomic inequalities, family structure, and health literacy remain universal challenges to ECC prevention. Although maternal education was not associated with caries experience, its association with pufa scores may indicate delayed care-seeking behaviors as disease severity progresses (6, 13).

This national dataset thus contributes valuable insight into the global discourse on ECC by providing evidence from a high-income country with universal access to healthcare but persisting high dental caries levels. The coexistence of accessible dental services with widespread untreated caries highlights that the availability of care alone is insufficient without effective early prevention, caregiver education, and community-based behavioral interventions (28). The findings therefore have direct implications for child oral health programs internationally, supporting the WHO and FDI calls for integrated, prevention-focused approaches to reduce the global burden of untreated caries (28, 33).

The study has some limitations. The cross-sectional design restricts causal interpretation of the relationships between ECC severity, OHRQoL, and demographic factors. Despite its nationally representative sample, the exclusion of absent or non-consenting participants may have introduced selection bias. Dental caries was assessed visually without radiographs, in accordance with WHO field protocols (17), which may have underestimated early lesions. Additionally, caregiver-reported OHRQoL data may have been affected by recall or response bias. Minor inter-examiner variability is also inevitable in large-scale epidemiologic surveys despite high calibration levels.

Nonetheless, this study provides nationally representative data from a large sample of preschool children across all six governorates of Kuwait, thereby enhancing the generalizability of the findings to Kuwaiti children attending public kindergartens. These data contribute to the global understanding of early childhood caries as a chronic, behaviorally influenced condition with wide-ranging effects on oral health and quality of life. However, caution is warranted when extrapolating the results to non-Kuwaiti populations or children enrolled in private schools, where socioeconomic and healthcare access factors may differ. The study findings highlight the urgent global need for early, preventive, and family-centered approaches to oral health promotion in preschool children.

## Conclusion

In conclusion, early childhood caries had a considerable negative impact on the oral health-related quality of life of preschool children and their families. Caries severity was associated with poorer child oral health-related quality of life but showed no additional impact at the family level. Dental pain and eating difficulties were the most frequent child impacts, while parental guilt and work disruption were commonly reported by caregivers. These findings underscore the importance of early preventive strategies and family-centered oral health programs to reduce the burden of ECC and improve child and family well-being.

## Data Availability

The original contributions presented in the study are included in the article/Supplementary Material, further inquiries can be directed to the corresponding author.
